# Inequalities in maternal health service utilisation in Somalia: analysis of the 2020 Somali health and demographic survey

**DOI:** 10.1080/16549716.2026.2712181

**Published:** 2026-08-05

**Authors:** Jamila Aden, Delia Hendrie, Judith Daire, Daniel Gashaneh Belay, Jennifer Dunne

**Affiliations:** a Deanship of Graduate Studies and Research, East Africa University, Bosaso, Puntland, Somalia; b Curtin School of Population Health, Curtin University, Perth, Western Australia, Australia; c enAble Institute, Curtin University, Perth, Western Australia, Australia

**Keywords:** Maternal health, health service utilisation, health equity, universal health coverage, Somalia

## Abstract

**Background:**

Somalia has one of the highest maternal mortality ratios and low coverage of maternal health services. Evidence on socioeconomic and geographic inequalities across maternal care continuum remains limited.

**Objective:**

This study examined wealth-related inequalities and determinants of maternal health service utilisation in Somalia.

**Methods:**

We analysed data from the 2020 Somali Health and Demographic Survey (SHDS), including 8,598 ever-married women aged 15–49 years with a recent live birth. Outcomes were at least one antenatal care visit (ANC), health facility childbirth, and postnatal care (PNC) within 2 days of birth. Multilevel mixed-effects logistic regression examined associations with socioeconomic, geographic, and empowerment-related factors, while Erreygers Concentration Indices (ECIs) assessed wealth-related inequalities. Results are presented as adjusted odds ratios (aORs), ECIs, and 95% confidence intervals (CIs).

**Results:**

Coverage was low: 32.4% attended at least one ANC visit, 24.2% delivered in a health facility, and 10.9% received PNC. Nomadic women were less likely to deliver in a health facility (aOR = 0.45; 95% CI: 0.35, 0.58) and receive early PNC (aOR = 0.49; 95% CI: 0.32, 0.75). Rural residence, the Northeastern and Southcentral zones, and higher parity were associated with lower service utilisation, whereas higher education, mobile phone ownership, household wealth, and media exposure increased utilisation. Pro-rich inequalities were observed for ANC (ECI: 0.40), health facility childbirth (ECI: 0.38), and early PNC (ECI: 0.19).

**Conclusions:**

Maternal health service utilisation remains critically low with socioeconomic and geographic inequalities. Equity-focused strategies targeting rural, remote, and nomadic populations are needed to accelerate universal health coverage.

## Paper Context

**Main findings**: Coverage of antenatal care, facility childbirth, and early postnatal care in Somalia remains critically low, with substantial inequalities by wealth, geography, and community type.

**Added knowledge**: Using nationally representative survey data, this study shows that women from nomadic, and poorer remote communities are consistently less likely to access essential maternal health services across the maternal care continuum.

**Global health impact for policy and action**: Reducing maternal health inequalities in fragile settings will require targeted outreach, financial protection, stronger primary health care, and sustained investment in women’s education and empowerment.

## Background

Despite notable improvement in maternal survival between 2000 and 2015, progress plateaued during the early years of the Sustainable Development Goal (SDG) period [1]. By 2023, the global maternal mortality ratio (MMR) was 197 deaths per 100 000 livebirths [2], indicating that the world is not on track to achieve SDG target 3.1, which aims to reduce the global MMR to less than 70 per 100 000 livebirths by 2030 [1]. Although the global MMR has declined overall, it conceals significant regional disparities, particularly in sub-Saharan Africa (SSA), which accounted for approximately 70% of global maternal deaths in 2020 [3,4]. In this region, the MMR fell from 802 to 536 deaths per 100,000 live births between 2000 and 2020, yet it remains more than twice the global average. Consequently, none of the SSA countries are projected to achieve the global benchmark by 2030 [4]. Persistent disparities in maternal mortality ratios between wealthy and poor populations highlight deep inequalities in the quality of maternal health care [4]. These regional and socioeconomic variations in MMR are further reflected in substantial differences in the lifetime risk of maternal deaths [3], underscoring that maternal mortality is not only a major public health crisis but also a critical indicator of national development, reflecting entrenched social and economic inequities between nations [3].

Such inequalities are most acute in fragile settings like Somalia, a low-income country with one of the highest MMR of 692 deaths per 100,000 live births in 2020 [5], where recurrent disease outbreaks, ongoing conflict, and frequent public health emergencies exacerbate systemic weakness. These challenges heighten pregnancy-related risks, disrupting already weak health infrastructure and restricting access to essential maternal care services such as antenatal care (ANC), facility delivery, and postnatal care (PNC) [6]. Beyond these structural barriers, individual and community-level determinants strongly influence maternal health service utilisation. In Somalia, women’s use of maternal health services and their choice of delivery location are shaped by socio-economic characteristics, educational attainment, geographical location, women’s autonomy, cultural norms, quality of services, and physical accessibility to health facilities, particularly the distance to care and the availability of transportation [7–10].

However, empirical evidence from Somalia remains sparse. Recent analysis using Somalia’s SHDS data has advanced understanding of maternal health service utilisation but is mostly descriptive, focusing on poverty determinants [11] or individual service component [12] such as delivery location alone [13,14]. Other studies have examined service coverage patterns rather than socioeconomic and regional determinants [14,15] or have been confined to subnational analysis, limiting generalisability [11,14]. Consequently, little is known about how socioeconomic status, geography, and women’s empowerment jointly influence maternal health service utilisation care within national representative framework. This study addresses this evidence gap identifying the social, economic, and geographic determinants of three maternal health outcomes: (i) antenatal care (ANC), (ii) facility delivery, and (iii) early PNC (within 2 days) using SHDS 2020 data. The findings will provide evidence on how socioeconomic status, regional disparities, and women’s empowerment jointly shape inequalities in maternal service utilisation in Somalia, offering policy-relevant insights for advancing national health goals and global commitments under SDG 3.1 (maternal mortality reduction) and SDG 5 (gender equality and women’s empowerment).

## Methods

### Data source, study setting, and period

This study utilised data from the Somali Health and Demographic Survey (SHDS 2020), a nationally representative, cross-sectional household survey conducted by the Federal Government of Somalia in collaboration with international partners under the Demographic and Health Survey (DHS) program hereafter referred to as SHDS [5]. The SHDS collected detailed information on fertility, maternal and child health, nutrition, mortality, and health-seeking behaviour. Somalia, located in the Horn of Africa and bordered by Djibouti, Ethiopia, Kenya, the Gulf of Aden and the Indian Ocean, covers approximately 637,657 km^2^ and had an estimated population of 18.4 million in 2023 [12]. The population is predominantly ethnic Somali, and Mogadishu functions as the political and economic hub. The country’s arid to semi-arid climate supports agriculture and livestock production; however, persistent insecurity and limited public investment have constrained access to healthcare. Somalia continues to experience high maternal and child mortality, malnutrition, and micronutrient deficiencies, with health service delivery heavily reliant on humanitarian organisations [15].

### Participants and sample size

The SHDS collected data from all 18 pre-war regions of Somalia. Each region was stratified into urban, rural, and nomadic domains, except Banadir (entirely urban), producing 55 initial strata. Security limitations led to the exclusion of several strata from Lower Shabelle, Middle Juba, and Bay, resulting in 47 final strata. Sampling followed a three-stage stratified cluster design: (1) selection of Primary Sampling Units (PSUs) proportional to population size; (2) selection of Secondary Sampling Units (SSUs); and (3) systematic sampling of 30 households per cluster.

**Figure 1.**
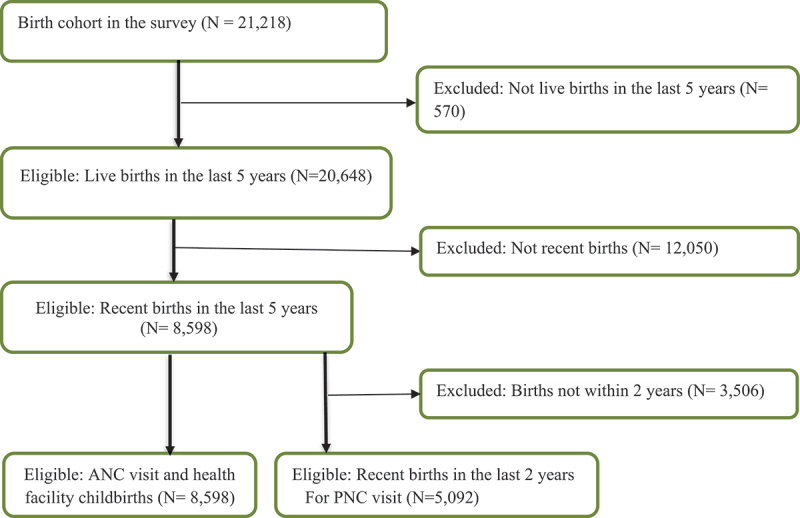
Selection of eligible women aged 15–49 years using the Somalia health and demographic survey 2020.

The survey initially included a birth cohort of 21,218 women. Of these, 570 were excluded because they did not have a live birth in the preceding 5 years, leaving 20,648 eligible women with at least one live birth during the reference period. Among these, 12,050 births were excluded because the analysis was restricted to the most recent live birth reported within the 5 years preceding the survey, thereby avoiding multiple observations per women. This resulted in 8,598 women with live births in the last 5 years who were eligible for analyses of antenatal care (ANC) visits and health facility childbirth. For the assessment of postnatal care (PNC) visits, we further restricted the sample to births occurring within the last 2 years, excluding 3,506 births that fell outside this period, yielding a final analytic sample of 5,092 women. Figure 1 presents the flow of participant selection from the full SHDS sample to the final study population.

### Outcome variables

Three maternal health outcomes were analysed: (1) Antenatal care (ANC) utilisation, defined as receipt of at least one ANC visits during the most recent pregnancy, recoded as *yes if they have at least one ANC visit, otherwise not*. (2) Health facility childbirth, defined as the place of delivery for the most recent birth, categorised as health facility (birth at a public or private health facility) versus non-institutional delivery (birth at home or other non-health facility location). (3) Early postnatal care (PNC), defined as the receipt of a postnatal check-up for the mother within 2 days of delivery for the most recent birth, categorised as either yes or no (Supplementary Table 1). Although the World Health Organization recommends a minimum of four ANC visits and more recently, eight contacts during pregnancy [16], a threshold of at least one visit (ANC1^+^) was used because higher order ANC indicators contained substantial missing or implausible values in the survey data. In many low- and middle-income settings, initiation of at least one ANC visit represents a critical first point of contact with formal maternal health care and an important entry point into the continuum of maternal care.

### Independent variables

Potential determinants were selected based on prior literature and data availability in the SHDS. They encompassed demographic, socioeconomic, geographic, behavioural, and empowerment domains (Supplementary Table 1). Key variables included maternal age (15–19, 20–29, 30–39, 40–49 years), marital status (married, divorced, widowed), education level (no education, primary, secondary/higher), household wealth quintile (lowest, second, middle, fourth, highest), residence type (urban, rural, nomadic), access to media defined as listening to radio or watching television (≥once a week, <once a week, not at all), and technology access defined as ownership of mobile phone (yes/no). Women’s empowerment is defined as married or partnered women who participate in all decision-making items and disagree with all justifications for wife-beating [17]. Participation in decision-making is operationalised as currently married women usually making all three specific decisions either alone or jointly with their husband: on their own health care, on large household purchases, and regarding visits to family or relatives. Similarly, attitudes toward wife-beating are operationalised as women who disagreed that a husband is justified in hitting or beating his wife for all the reasons: going out without telling him; neglecting the children; arguing with him; refusing to have sexual intercourse with him; and burning food. Women’s empowerment defined as participation in all the three household decision-making (yes/no) and disagreement with all the five reasons of justifying wife beating (yes/no) (Supplementary Table 1).

### Data management and analysis

All analyses incorporated the SHDS two-stage stratified cluster design and sampling weights to produce nationally representative estimates. Data were analysed using Stata 17 software. Descriptive statistics reported weighted frequencies and percentages. To assess multicollinearity among covariates, Variance Inflation Factors (VIFs) were examined. Variables with VIFs greater than 10 were considered for exclusion or recoding to minimise collinearity.

### Statistical model of analysis

Determinants of ANC, health facility childbirth and PNC visit were evaluated using multilevel mixed-effect binary logistic regression. Separate models were fitted for each outcome, including all determinant variables simultaneously. This approach estimates adjusted associations while accounting for potential confounding among determinants. A multilevel mixed-effects binary logistic regression was conducted to examine factors associated with ANC visits, place of delivery, and early PNC visits among women, accounting for the hierarchical structure of the SHDS data (women nested within the clusters). The cluster corresponds to the Primary Sampling Unit (PSU) in SHDS, represented by the random variable ‘V001’. Each cluster is a georeferenced unit comprising a grouping of households, typically about 25–30 households selected to participate in the survey [5]. Model 1 (null model) was fitted with only the outcome variable and a cluster-level random intercept (V001), without including any independent variables, to quantify baseline between-cluster variability and compute measures such as the intra-class correlation coefficient (ICC), median odds ratio (MOR), and proportional change in variance (PCV). Model 2 incorporated a cluster-level random intercept and included individual-level covariates to examine their association with the outcome while accounting for clustering. Model 3 included a cluster-level random intercept with community-level explanatory variables to assess the influence of contextual factors on the outcomes. The final model (Model 4) simultaneously included individual- and community-level explanatory variables, together with a cluster-level random intercept, to assess their combined effects on the outcome while accounting for clustering (Supplementary Table 2–4).

### Random effects analysis

The measure of variation or random effects was estimated using the intra-class correlation coefficient (ICC), the median odds ratio (MOR), and the proportional change in variance (PCV). The intra-class correlation coefficient (ICC) quantifies the proportion of total variation in maternal health service utilisation that is attributable to differences between clusters, thereby indicating the degree of clustering in the data. A higher ICC indicates stronger clustering, meaning that women within the same cluster are more similar in their service utilisation than those in different clusters. This justifies the use of multilevel modelling, as it accounts for the hierarchical structure of the data and the non-independence of observations within clusters [18–20].

The median odds ratio (MOR) is the median of the odds ratios between a higher risk and a lower risk cluster when two clusters are randomly selected. It represents the median increase in the odds of the outcome when moving from a lower risk cluster to a higher risk cluster, assuming two clusters are randomly selected. An MOR value of 1 indicates no variation between clusters, while higher values indicate greater unexplained heterogeneity at the community level [18–20].

The proportional change in variance (PCV) quantifies the proportion of between-cluster variation in maternal health service utilisation that is explained by the inclusion of individual- and community-level factors relative to the null model. Higher PCV values suggest that the included variables explain a greater proportion of the contextual variability.

### Model fitness tests

Since the models were nested, model comparison was performed using the deviance test (−2*log-likelihood). The model with the lowest deviance was selected as the best-fitting model for each maternal health service. Accordingly, Model 4 exhibited the lowest deviance across all outcomes and was therefore chosen as the final model. Women’s individual sample weights were applied to adjust for the complex survey design, and the results are presented as odds ratios.

We assessed wealth-related inequalities in maternal health service utilisation using Erreygers Concentration Index (ECI), which is appropriate for binary health outcomes and accounts for the bounded nature of the variables. Household wealth status was measured using the SHDS wealth index, which was principal component-analysed, and ranked from the poorest to the richest quintile. The ECI was estimated for antenatal care (ANC) visit, health facility childbirth, and early postnatal care (PNC) visits using sampling weights to account for the complex survey design. Positive ECI values indicate a disproportionate concentration of service utilisation among wealthier women (pro-rich inequality), whereas negative values indicate a concentration among poorer women (pro-poor inequality).

## Results

A total of 8,598 (weighted 8500) reproductive-age women were included in this study. Nearly half (49%) of the women were between 20 and 29 years old, with a median age of 28.8 years (interquartile range (IQR) = 10 years). More than four-fifths (83.2%) of women had no formal education, and 88% of women had two or more births. The women were distributed nearly evenly across the five wealth index categories. One-third (29.6%) of women lived in nomadic settings, while regional representation shows that more than 45.2% of women reside in the South-Central zone. Most women (83.8%) reported no exposure to radio or television, and 81.7% of women reported owning a telephone. Moreover, 14.3% of women were empowered (Table 1).

**Table 1.** Sociodemographic characteristics of women from the Somali health and demographic survey 2020 (*n* = 8,598).

Variables	Categories	Frequency	Percentage
**Socio-demographic variables**			
Age of women (years)	15–19	567	6.6
	20–29	4,194	48.8
	30–39	3,152	36.7
	40–49	685	8.0
Educational attainment of women	No education	7,157	83.2
	Primary	1,039	12.1
	Secondary/above	402	4.7
Marital status of the mother	Currently Married	7,797	90.7
	Divorced	559	6.5
	Widowed	242	2.8
Wealth index	Lowest	1,852	21.5
	Low	1,887	22.0
	Middle	1,692	19.7
	High	1,678	19.5
	Highest	1,489	17.3
Media access	Not at all	7,208	83.8
	<Once a week	395	4.6
	≥Once a week	995	11.6
Parity	Primiparous	1,028	12.0
	Multiparous	3,672	42.7
	Grand Multiparous	3,896	45.3
Owns a mobile telephone	Yes	7,028	81.7
	No	1,570	18.3
Women empowerment	No	5,241	85.7
	Yes	873	14.3
**Community-level variables**			
Residence	Urban	3,675	42.7
	Rural	2,375	27.6
	Nomadic	2,548	29.6
Regions	Northwestern Zone	3,280	38.1
	Northeastern Zone	1,433	16.7
	Southcentral Zone	3,885	45.2

### Maternal health care service use among women in Somalia

**Figure 2.**
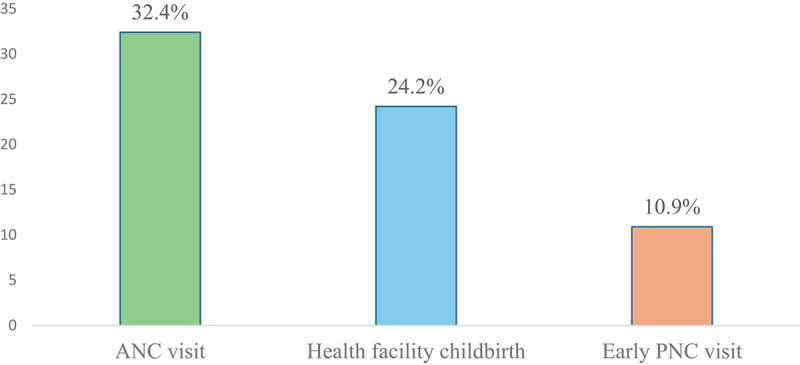
Maternal health care service use among women in aged 15–49 years in Somalia, based on the 2020 Somalia health and demographic survey.

In our study, only 32.4% (95% CI: 31.4, 33.4) of women attended at least one ANC visit during their pregnancy. Less than one quarter, 24.2% (95% CI: 23.3, 25.1) of women delivered in a health facility. Early postnatal care (PNC) was received by only 10.9% (95% CI: 10.1–11.8) of women (Figure 2).

### Determinants of maternal health care service use among women in Somalia

The random-effects analysis revealed substantial clustering in maternal health service utilisation, underscoring the importance of applying a multilevel mixed-effects modelling approach to account for between-cluster variability. The ICC results from the Model 1 analysis of ANC visit, childbirth service, and PNC service outcomes reveal that 41%, 51%, and 34% of the variation in these maternal health services was due to clustering. Furthermore, the proportional change in variance (PCV) in Model 4 showed that the inclusion of both individual- and community-level variables reduced the cluster-level variance by 68.86%, 70.71%, and 71.89% for ANC visits, childbirth service utilisation, and PNC service use, respectively, compared with the null model (Table 2).

**Table 2.** Random effect and model comparison for multilevel models of maternal health care service use among women in Somalia.

Outcome variable and model parameters		Null model (Model 1)	Model 2	Model 3	Model 4
**ANC visits**					
Random effect	Cluster level variance (σ2)	2.28 (1.79, 2.91)	0.85 (0.62, 1.16)	2.11 (1.65, 2.70)	0.71 (0.52, 0.97)
	ICC	0.41 (0.36, 0.47)	0.21 (0.16, 0.26)	0.39 (0.33, 0.46)	0.18 (0.14, 0.23)
	MOR	4.23 (3.59, 5.08)	2.54 (2.16, 2.97)	4.06 (3.45, 4.85)	2.23 (1.92, 2.62)
	PCV	Ref	62.72%	7.43%	68.86%
Model fitness test	Log likelihood	−4697.97	−4449.19	−4672.10	−4418.03
	Deviance	9395.94	8898.38	9344.20	8836.06
	Mean VIF	—	2.13	1.98	1.96
**Health facility childbirth**					
Random effect	Cluster level variance (σ^2^)	3.38 (2.63, 4.36)	1.09 (0.79, 1.49)	3.37 (2.59, 4.37)	0.99 (0.72, 1.37)
	ICC	0.51 (0.45, 0.57)	0.25 (0.20, 0.30)	0.51 (0.44, 0.57)	0.23 (0.18, 0.29)
	MOR	5.78 (4.69, 7.30)	2.70 (2.23, 3.33)	5.88 (4.76, 7.43)	2.59 (2.15, 3.19)
	PCV	Ref	67.75%	0.30%	70.71%
Model fitness test	Log likelihood	−4010.44	−3693.07	−3965.59	−3654.78
	Deviance	8020.88	7386.15	7931.18	7309.55
	Mean VIF	—	2.13	1.98	1.96
**PNC visits**					
Random effect	Cluster level variance (σ^2^)	1.70 (1.17, 2.48)	0.52 (0.30, 0.89)	1.65 (1.13, 2.40)	0.48 (0.28, 0.83)
	ICC	0.34 (0.26, 0.43)	0.14 (0.08, 0.21)	0.33 (0.26, 0.42)	0.13 (0.08, 0.20)
	MOR	3.47 (2.80, 4.44)	1.98 (1.56, 2.63)	3.40 (2.73, 4.33)	1.91 (1.51, 2.52)
	PCV	Ref	69.74%	3.14%	71.89%
	Log likelihood	−1700.35	−1525.38	−1683.36	−1514.30
	Deviance (−2LL)	3400.71	3050.77	3366.72	3028.60
	Mean VIF	—	2.13	1.98	1.96

Table 3 presents the selected model (Model 4) for each of the three maternal health services. Based on this, women’s educational attainment, household wealth index, parity, media access, residence, and region were significant determinants of maternal health service utilisation (Table 3). Women with secondary or higher education had significantly higher odds of attending antenatal care (ANC) (AOR = 2.28; 95% CI: 1.76, 2.94), delivering at a health facility (AOR = 2.58; 95% CI: 1.99, 3.35), and receiving postnatal care (PNC) (AOR = 2.60; 95% CI: 1.83, 3.69) compared with women with no formal education.

**Table 3.** Model 4 output of the multilevel mixed-effect binary logistic regression analysis of factors associated with maternal health care service use among women in Somalia.

Variables	Categories	ANC visit AOR (95% CI)	Health facility childbirth AOR (95% CI)	PNC visit AOR (95% CI)
**Socio-demographic variables**				
Age of women (years)	15–19	Ref.	Ref.	Ref.
	20–29	1.25 (0.98, 1.59)	1.06 (0.81, 1.39)	2.24 (1.48, 3.40) ***
	30–39	1.02 (0.78, 1.33)	1.18 (0.87, 1.59)	2.63 (1.64, 4.22)***
	40–49	0.82 (0.59, 1.13)	1.02 (0.71, 1.47)	2.82 (1.43, 5.56)**
Educational attainment of women	No education	Ref.	Ref.	Ref.
	Primary	1.92 (1.64, 2.25)***	1.79 (1.52, 2.10)***	1.83 (1.43, 2.34)***
	Secondary/above	2.28 (1.76, 2.94)***	2.58 (1.99, 3.35)***	2.60 (1.83, 3.69)***
Marital status of the mother	Currently Married	Ref.	Ref.	Ref.
	Divorced	0.73 (0.59, 0.90)**	1.14 (0.91, 1.43)	0.79 (0.51, 1.23)
	Widowed	0.70 (0.50, 1.00)	1.44 (0.99, 2.05)*	1.98 (0.99, 3.98)
Wealth index	Lowest	Ref.	Ref.	Ref.
	Low	1.37 (1.11, 1.68)**	1.91 (1.45, 2.53)***	1.53 (0.97, 2.39)
	Middle	2.15 (1.72, 2.68)***	4.02 (3.01, 5.37)***	2.34 (1.49, 3.66)***
	High	3.27 (2.62, 4.07)***	6.27 (4.71, 8.34)***	3.82 (2.51, 5.82)***
	Highest	3.56 (2.80, 4.52)***	9.70 (7.20, 13.06)***	5.83 (3.80, 8.95)***
Media access	Not at all	Ref.	Ref.	Ref.
	<Once a week	1.29 (1.02, 1.64)*	1.37 (1.07, 1.76)*	1.39 (0.97, 2.01)
	≥Once a week	1.92 (1.60, 2.31)***	1.34 (1.10, 1.63)**	1.79 (1.34, 2.39)***
Owns a mobile telephone	No	Ref.	Ref.	Ref.
	Yes	1.48 (1.25, 1.74)***	1.40 (1.15, 1.70)**	1.58 (1.11, 2.24)*
Parity	Primiparous	Ref.	Ref.	Ref.
	Multiparous	0.82 (0.68, 1.00)	0.69 (0.56, 0.84)***	0.51 (0.38, 0.68)***
	Grand multiparous	0.96 (0.78, 1.19)	0.53 (0.42, 0.66)***	0.36 (0.25, 0.52)***
Women empowerment	No	Ref.	Ref.	Ref.
	Yes	0.87 (0.74, 1.04)	0.96 (0.79, 1.15)	1.39 (1.00, 1.82)
**Community-level variables**				
Residence	Urban	Ref.	Ref.	Ref.
	Rural	0.82 (0.69, 0.97)*	0.68 (0.56, 0.82)***	0.77 (0.59, 1.02)
	Nomadic	0.98 (0.80, 1.20)	0.45 (0.35, 0.58)***	0.49 (0.32, 0.75)**
Regions	Northwestern Zone	Ref.	Ref.	Ref.
	Northeastern Zone	0.65 (0.53, 0.78)***	0.58 (0.47, 0.73)***	0.67 (0.48, 0.94)*
	Southcentral Zone	0.55 (0.46, 0.65)***	0.49 (0.40, 0.59)***	0.56 (0.42, 0.74)***

*p-value < 0.05. **p-value < 0.01. AOR: Adjusted odds ratio. CI: Confidence interval, ANC: Antenatal care, PNC: Postnatal care visit.

Household wealth showed a strong positive gradient with service utilisation. Women in the highest wealth quintile had markedly higher odds of ANC attendance (AOR = 3.56; 95% CI: 2.80, 4.52), facility delivery (AOR = 9.70; 95% CI: 7.20, 13.06), and PNC utilisation (AOR = 5.83; 95% CI: 3.80, 8.95) compared with those in the lowest quintile. Similarly, women who accessed media at least once per week were more likely to utilise services, with higher odds of ANC attendance (AOR = 1.92; 95% CI: 1.60, 2.31), facility delivery (AOR = 1.34; 95% CI: 1.10, 1.63), and PNC utilisation (AOR = 1.79; 95% CI: 1.34, 2.39) compared to those with no media exposure.

At the community level, women residing in rural areas had lower odds of utilising ANC (AOR = 0.82; 95% CI: 0.69, 0.97) and of delivering at a facility (AOR = 0.68; 95% CI: 0.56, 0.82) compared to urban resident women. Moreover, women living in nomadic settings had substantially lower odds of facility delivery (AOR = 0.45; 95% CI: 0.35, 0.58) and PNC utilisation (AOR = 0.49; 95% CI: 0.32, 0.75) than urban residents.

Compared with women residing in the Northwestern Zone, those in the Northeastern Zone had lower odds of ANC attendance (AOR = 0.65; 95% CI: 0.53, 0.78), facility delivery (AOR = 0.58; 95% CI: 0.47, 0.73), and PNC utilisation (AOR = 0.67; 95% CI: 0.48, 0.94). Similarly, women in the Southcentral Zone had significantly reduced odds of ANC attendance (AOR = 0.55; 95% CI: 0.46, 0.65), facility delivery (AOR = 0.49; 95% CI: 0.40, 0.59), and PNC utilisation (AOR = 0.56; 95% CI: 0.42, 0.74) relative to those in the Northwestern Zone (Table 3).

## Wealth-related inequalities in maternal health service use

Substantial wealth-related inequalities were observed across all maternal health services, with utilisation disproportionately concentrated among women from wealthier households. The Erreygers Concentration Index (ECI) was positive for all three services, indicating pro-rich inequality: antenatal care (ANC) visits (ECI = 0.40, 95% CI: 0.37, 0.44), health facility childbirth (ECI = 0.38, 95% CI: 0.35, 0.45), and early postnatal care (PNC) visits (ECI = 0.19, 95% CI: 0.16, 0.22) (Table 4).

**Table 4.** Wealth-related inequalities in maternal health service use.

Maternal health service	Erreygers Concentration index (ECI)	P- value
ANC visit	0.40 (0.37, 0.44)	<0.001
Health facility childbirth	0.38 (0.35, 0.45)	<0.001
Early PNC visit	0.19 (0.16, 0.22)	<0.001

**Figure 3.**
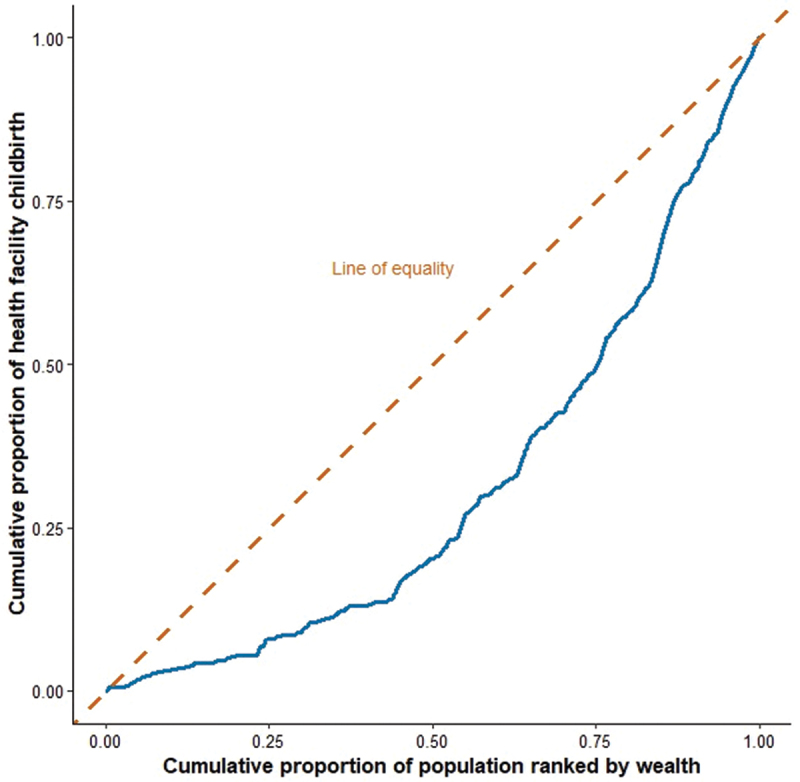
Wealth-related inequalities in childbirth at health facilities among women in Somalia.

The corresponding concentration curves in all the three maternal health services lay below the line of equality, confirming that utilisation of antenatal care (ANC), health facility childbirth, and early postnatal care (PNC) was disproportionately concentrated among women from wealthier households. The greatest deviation from the line of equality was observed for ANC, followed closely by health facility childbirth (Figure 3 and Supplementary Figures 1 and 2).

## Discussion

Using nationally representative SHDS 2020 data, this study examined how socioeconomic status, geography, and women’s empowerment are associated with the utilisation of three essential maternal health services in Somalia: at least one ANC visit, health facility delivery, and early PNC within 2 days of birth. Overall coverage was critically low across all three indicators, and marked inequalities were evident. Higher education, household wealth, and information access (media exposure and mobile phone ownership) were consistently associated with greater service use, whereas nomadic residence and living outside the Northwestern Zone were associated with lower utilisation, even after accounting for individual and community-level factors. These findings indicate that current maternal health strategies are insufficiently reaching the most disadvantaged women and underscore the need for equity-focused approaches that prioritise underserved regions, nomadic populations, and women with limited socioeconomic resources to improve maternal survival and accelerate progress towards universal health coverage.

Coverage of maternal health services in Somalia remains substantially lower than regional and global benchmarks. In this study, only around one-third of women attended at least one antenatal care (ANC) visit during pregnancy, approximately one-quarter delivered in a health facility, and roughly one in 10 received early postnatal care within 2 days of birth. According to the SHDS 2020, only about 7% of women completed four or more ANC visits, far below the levels typically reported across low- and middle-income countries [5]. In many such settings, approximately half of pregnant women attend at least four ANC visits, and regional estimates for sub-Saharan Africa are substantially higher [7,8]. Even in resource-constrained countries such as Uganda and Ethiopia, reported ANC4^+^ coverage has been around 60% and 32%, respectively [21]. These comparisons highlight the magnitude of Somalia’s maternal health service gap and emphasise the need for urgent improvements in access across the maternal care continuum.

The persistently low utilisation of maternal health services in Somalia reflects the broader fragility of the national health system. Decades of conflict, political instability, and chronic underinvestment in public health infrastructure have limited the availability and accessibility of maternal care services [22]. Weak governance, shortages of skilled health personnel, and inadequate health facility coverage further constrain service delivery, particularly in rural and remote areas [9]. Travel distances, poor road networks, insecurity, and limited transportation options also impede timely access to care [6]. Together, these factors contribute to persistently low service utilisation and reinforce existing inequalities in maternal health outcomes.

Socioeconomic disparities emerged as a central determinant of maternal health service utilisation. Women with higher levels of education, greater household wealth, and access to information through media or mobile phones were significantly more likely to utilise antenatal, delivery, and postnatal services. These findings are consistent with a large body of evidence showing that socioeconomic status strongly influences access to maternal health services in low- and middle-income countries [7,8]. Higher educational attainment was associated with greater utilisation of maternal health services. This association may reflect differences in health literacy and awareness of pregnancy-related risks and ability to navigate health systems. Higher household wealth was greater maternal service utilisation and may reflect reduced financial barriers to accessing care. The magnitude of the wealth gradient observed in this study was particularly notable. Women in the highest wealth quintile had almost 10 times greater odds of delivering in a health facility compared with women in the poorest wealth quintile. This finding highlights the persistence of substantial financial and socioeconomic barriers to accessing skilled maternity care and suggests that economic inequalities remain a major challenge to achieving equitable maternal health coverage in Somalia. The concentration index analyses further confirmed substantial pro-rich inequalities across the maternal care continuum. Utilisation of antenatal care, health facility childbirth and early postnatal care were disproportionately concentrated among wealthier women, indicating that improvements in service coverage have not been equitably distributed across socioeconomic groups. These findings suggest that progress towards universal health coverage in Somalia remains constrained by persistent economic inequalities in access to essential maternal health services. Previous studies conducted in Somalia have similarly shown that women’s economic status, educational attainment, and geographic location are key determinants of maternal health service utilisation, highlighting the importance of financial resources and maternal health literacy in enabling access to skilled birth attendance and other essential services [23,24].

Geographic inequalities were also evident in this study. Women residing in nomadic areas and those living in the southeastern and south-central zones were significantly less likely to use maternal health services compared with women living in the urban Northwestern Zone. These findings are consistent with previous analyses indicating that maternal health coverage has historically been higher in the Northwestern Zone than in other parts of Somalia [25]. Differences in regional utilisation may be associated with broader contextual factor, including variation in governance, health system capacity, infrastructure, and security conditions across the country, although these factors were not directly measured in this study. The Northwestern region has experienced relatively greater political stability and administrative capacity, which may have supported more sustained investments in maternal health services. In contrast, regions affected by insecurity and weaker governance structures often face shortages of health facilities, limited infrastructure, and reduced access to skilled health providers. Longer travel distances, poor road networks, and security concerns can further restrict access to care, particularly for women living in rural or nomadic communities [6].

Interestingly, women’s empowerment indicators were not significantly associated with maternal health service utilisation in this study. In other settings, women’s autonomy and decision-making power have been shown to increase the likelihood of attending antenatal care and delivering in health facilities [26]. One possible explanation for the absence of a similar association in the present analysis is that structural and health system constraints may outweigh the influence of individual-level autonomy in fragile settings such as Somalia [9]. When services are limited or geographically inaccessible, even women with greater decision-making power may face substantial barriers to accessing care. This finding suggests that improvements in maternal health service utilisation in Somalia may depend more strongly on addressing systemic access barriers than on changes in individual-level empowerment alone.

Our findings are also consistent with evidence from other fragile and conflict-affected settings in sub-Saharan Africa, where maternal health service utilisation is frequently constrained by weak health systems, poverty, insecurity, limited awareness of maternal health care, and long distances to health facilities [7,27–30]. Evidence from our recent scoping review similarly indicates that although some progress has been made in increasing maternal health service coverage in low- and middle-income countries, substantial inequities persist, particularly among socioeconomically disadvantaged and geographically marginalised populations [10]. These patterns highlight the continuing challenge of ensuring equitable access to maternal health services in fragile contexts.

### Strengths and limitations

This study has several important strengths. To our knowledge, it represents one of the few national-level analyses in Somalia to examine antenatal care, health facility delivery, and early postnatal care simultaneously within a unified analytic framework, allowing inequalities to be assessed across the maternal health care continuum. The use of nationally representative SHDS data enhances the generalisability of the findings, while the application of survey-weighted multilevel modelling enabled the study to account for both individual and community-level determinants of maternal health service utilisation. In addition, the inclusion of women’s empowerment indicators alongside socioeconomic and geographic variables provides a more comprehensive assessment of the factors influencing maternal health care use in a fragile and under-studied setting.

Several limitations should also be acknowledged. First, the cross-sectional design of the survey limits the ability to draw causal inferences from the observed associations. Second, the reliance on self-reported data may introduce recall or social desirability bias, although restricting the analysis of postnatal care to births within the previous 2 years helped minimise potential recall errors. Third, due to substantial missing and implausible values for higher order antenatal care indicators in the 2020 SHDS, ANC utilisation was assessed using attendance at least one antenatal care visit (ANC1^+^). Although ANC1^+^ captures initial contact with maternal health services, it does not reflect the adequacy or quality of antenatal care received.

Finally, security-related constraints during survey fieldwork resulted in the exclusion of some strata in a small number of regions. Because these areas are likely to represent some of the most underserved populations, the findings may underestimate the true magnitude of geographic inequalities in maternal health service utilisation.

### Policy implications

Maternal health service utilisation in Somalia remains critically low, with substantial inequities in access across socioeconomic and geographic groups. Without targeted interventions aimed at disadvantaged populations, improvements in national maternal health indicators are likely to remain limited. When large segments of the population face persistent barriers to care, progress concentrated among better-off groups alone is unlikely to substantially increase national coverage or reduce disparities [12,26].

Addressing these challenges will require equity-focused strategies that prioritise women living in remote, nomadic, and socioeconomically disadvantaged communities. Mobile and outreach clinics may help extend maternal health services to populations living far from fixed health facilities. Financial protection mechanisms, such as voucher schemes or targeted subsidies, could reduce cost-related barriers for poorer households. Community health workers may also play an important role in raising awareness of maternal health services, facilitating referrals, and strengthening links between communities and health facilities.

At the same time, broader health system strengthening will be essential to ensure sustainable improvements in maternal health service utilisation. Investments in health infrastructure, expansion of the skilled health workforce, and improvements in transportation and referral systems are critical for improving access to maternal care. Strengthening governance and coordination within the health system will also be necessary to ensure that services reach underserved regions and populations. In the context of the Sustainable Development Goals and global commitments to universal health coverage, addressing inequities in maternal health service utilisation is particularly important for Somalia. Increasing overall service coverage must be accompanied by deliberate efforts to reduce disparities and ensure that the most disadvantaged women are able to access essential maternal health services.

## Conclusions

Maternal health service utilisation in Somalia remains critically low, with substantial socioeconomic and geographic inequalities shaping access to care. Women who are poorer, less educated, and living in nomadic or underserved regions are significantly less likely to receive essential maternal health services across the continuum of care. Equity-focused policies that address both structural barriers and social determinants of health are essential for improving maternal health outcomes and accelerating progress toward universal health coverage and the Sustainable Development Goals. Sustained political commitment and targeted investment will be required to strengthen health system capacity and ensure that no woman is left behind.

## Supplementary Material

Supplementary File1 29062026.docx

## Data Availability

The dataset supporting the conclusions of this article is available from the Demographic and Health Survey (DHS) Program upon reasonable request and with permission from DHS Program (https://dhsprogram.com).
