# Supplementary material for: Inequalities in maternal health service utilisation in Somalia: analysis of the 2020 Somali health and demographic survey
Source: Glob Health Action. 2026 Aug 5;19(1):2712181. doi: 10.1080/16549716.2026.2712181 (PMC13446055; doi:10.1080/16549716.2026.2712181)
Supplement: Supplementary file 1 [file ZGHA_A_2712181_SM7467.docx]

**Supplementary Files**

Supplementary Table 1. Study variables and measurement in the study of the determinants of maternal health care service use among women in Somalia

| **Level** | **Variables** | **Measurements** |
| --- | --- | --- |
| Outcome variables | ANC visit | Whether a woman had at least one ANC visit during her pregnancy for her most recent birth |
|  | Health facility childbirth | Childbirth occurred in a public, private, or non-governmental organisation (NGO) affiliated with a health facility for her most recent birth. |
|  | Early PNC visit | Whether a woman received at least one PNC checkup by a health professional within 2 days of birth for her most recent birth. |
| Independent variables | Age of women | The age of mother/caregiver was categorised as 15-19, 20-29, 30-39 and 40-49. |
|  | Education level | The highest educational attainment of the women is categorised as not educated, primary, secondary or above. |
|  | Marital status | The marital status of women is categorised as currently married, divorced, or widowed |
|  | Media exposure | A composite variable obtained by combining whether a respondent reads listens to the radio, and watches television, with a value of “0” if a women were not exposed to at least one of the two media, and “1” if a woman has access/exposure to at least one of the three media. |
|  | Mobile telephone | Whether a woman has their own telephone or not |
|  | Wealth index | The datasets contained a wealth index created using principal components analysis, coded as follows: lowest, low, middle, high, and highest (poorest, poorer, middle, richer, and richest) in the DHS dataset, respectively. |
|  | Parity | The number of children born is categorised as primiparous (1), multiparous (2-4), and grand multiparous (5 or more). |
|  | Women empowerment | Married/partnered women who were categorised as *empowered* based on their report in participating in all decision-making processes and disagree with all reasons justifying wife-beating unless they are not empowered.  Participation in decision-making: currently married women usually make all three specific decisions either alone or jointly with their husband: a) Own health care, b) Large household purchases, c) Visits to family or relatives.  Attitude towards wife beating: women who disagree that a husband is justified in hitting or beating his wife for any reason. 1) Going out without telling him, 2) Neglecting the children, 3) Arguing with him, 4) Refusing to have sexual intercourse with him, and 5) Burning food. |
|  | Geographic residency | Urban or rural, based on where the household lives. |
|  | Geographic region | The Northwestern Zone comprises the five regions, including Awdal, Woqooyi Galbeed, Togdheer, Sool, and Sanaag. The Northeastern Zone includes three administrative regions: Bari, Nugaal, and Mudug. The South-Central Zone covers the remaining regions of the country, namely Galgaduud, Hiraan, Middle Shabelle, Banadir, Bay, Bakool, Gedo, and Lower Juba |

Supplementary Table 2. Multilevel mixed-effect binary logistic regression analysis of factors associated with ANC service use among women in Somalia (n = 8,598)

| **Variables** | **Categories** | **Model 2**  **AOR (95% CI)** | **Model 3**  **AOR (95% CI)** | **Model 4**  **AOR (95% CI)** |
| --- | --- | --- | --- | --- |
| **Individual-level variables** | |  |  |  |
| Age of women (years) | 15-20 | Ref. |  | Ref. |
|  | 20–29 | 1.26 (0.99, 1.61) |  | 1.25 (0.98, 1.59) |
|  | 30–39 | 1.06 (0.81, 1.38) |  | 1.02 (0.78, 1.33) |
|  | 40–49 | 0.87 (0.63, 1.20) |  | 0.82 (0.59, 1.13) |
| Educational attainment of women | No education | Ref. |  | Ref. |
|  | Primary | 1.93 (1.65, 2.26) *** |  | 1.92 (1.64, 2.25) *** |
|  | Secondary/above | 2.24 (1.73, 2.90) *** |  | 2.28 (1.76, 2.94) *** |
| Marital status of the mother | Currently Married | Ref. |  | Ref. |
|  | Divorced | 0.72 (0.58, 0.89) ** |  | 0.73 (0.59, 0.90) ** |
|  | Widowed | 0.67 (0.48, 0.95) * |  | 0.70 (0.50, 1.00) |
| Wealth index | Lowest | Ref. |  | Ref. |
|  | Low | 1.25 (1.02, 1.54) * |  | 1.37 (1.11, 1.68) ** |
|  | Middle | 1.84 (1.48, 2.29) *** |  | 2.15 (1.72, 2.68) *** |
|  | High | 2.84 (2.27, 3.54) *** |  | 3.27 (2.62, 4.07) *** |
|  | Highest | 3.30 (2.59, 4.20) *** |  | 3.56 (2.80, 4.52) *** |
| Media access | Not at all | Ref. |  | Ref. |
|  | < Once a week | 1.27 (1.00, 1.61) |  | 1.29 (1.02, 1.64) * |
|  | ≥ Once a week | 1.99 (1.65, 2.39) *** |  | 1.92 (1.60, 2.31) *** |
| Owns a mobile telephone | No | Ref. |  | Ref. |
|  | Yes | 1.45 (1.23, 1.71) *** |  | 1.48 (1.25, 1.74) *** |
| Parity | Primiparous | Ref. |  | Ref. |
|  | Multiparous | 0.81 (0.67, 0.98) * |  | 0.82 (0.68, 1.00) |
|  | Grand multiparous | 0.93 (0.75, 1.15) |  | 0.96 (0.78, 1.19) |
| Women empowerment | No | Ref. |  | Ref. |
|  | Yes | 0.89 (0.75, 1.05) |  | 0.87 (0.74, 1.04) |
| **Community-level variables** | |  |  |  |
| Residence | Urban |  | Ref. | Ref. |
|  | Rural |  | 0.77 (0.65, 0.91) ** | 0.82 (0.69, 0.97) * |
|  | Nomadic |  | 0.87 (0.70, 1.07) | 0.98 (0.80, 1.20) |
| Regions | Northwestern Zone |  | Ref. | Ref. |
|  | Northeastern Zone |  | 0.65 (0.53, 0.79) *** | 0.65 (0.53, 0.78) *** |
|  | Southcentral Zone |  | 0.57 (0.48, 0.68) *** | 0.55 (0.46, 0.65) *** |

**p-value < 0.05, ** p-value <0.01, ***p-value < 0.001. AOR: Adjusted Odds Ratio. CI: Confidence Interval, ANC: Antenatal care, PNC: Postnatal care visit. Model 1 (null model) is without independent variables and not specified here.*

Supplementary Table 3. Multilevel mixed-effect binary logistic regression analysis of factors associated with health facility childbirth service use among women in Somalia (n = 8,598)

| **Variables** | **Categories** | **Model 2**  **AOR (95% CI)** | **Model 3**  **AOR (95% CI)** | **Model 4**  **AOR (95% CI)** |
| --- | --- | --- | --- | --- |
| **Individual-level variables** | |  |  |  |
| Age of women (years) | 15-20 | Ref. |  | Ref. |
|  | 20–29 | 1.11 (0.85, 1.45) |  | 1.06 (0.81, 1.39) |
|  | 30–39 | 1.28 (0.95, 1.72) |  | 1.18 (0.87, 1.59) |
|  | 40–49 | 1.14 (0.79, 1.64) |  | 1.02 (0.71, 1.47) |
| Educational attainment of women | No education | Ref. |  | Ref. |
|  | Primary | 1.86 (1.58, 2.19) *** |  | 1.79 (1.52, 2.10) *** |
|  | Secondary/above | 2.55 (1.96, 3.30) *** |  | 2.58 (1.99, 3.35) *** |
| Marital status of the mother | Currently Married | Ref. |  | Ref. |
|  | Divorced | 1.13 (0.90, 1.41) |  | 1.14 (0.91, 1.43) |
|  | Widowed | 1.37 (0.96, 1.95) |  | 1.44 (0.99, 2.05) * |
| Wealth index | Lowest | Ref. |  | Ref. |
|  | Low | 1.68 (1.28, 2.21) *** |  | 1.91 (1.45, 2.53) *** |
|  | Middle | 3.34 (2.52, 4.43) *** |  | 4.02 (3.01, 5.37) *** |
|  | High | 5.34 (4.04, 7.06) *** |  | 6.27 (4.71, 8.34) *** |
|  | Highest | 8.88 (6.62, 11.91) *** |  | 9.70 (7.20, 13.06) *** |
| Media access | Not at all | Ref. |  | Ref. |
|  | < Once a week | 1.34 (1.05, 1.72) * |  | 1.37 (1.07, 1.76) * |
|  | ≥ Once a week | 1.38 (1.13, 1.67) ** |  | 1.34 (1.10, 1.63) ** |
| Owns a mobile telephone | No | Ref. |  | Ref. |
|  | Yes | 1.32 (1.09, 1.59) ** |  | 1.40 (1.15, 1.70) ** |
| Parity | Primiparous | Ref. |  | Ref. |
|  | Multiparous | 0.68 (0.56, 0.84) *** |  | 0.69 (0.56, 0.84) *** |
|  | Grand multiparous | 0.51 (0.40, 0.64) *** |  | 0.53 (0.42, 0.66) *** |
| Women empowerment | No | Ref. |  | Ref. |
|  | Yes | 1.00 (0.83, 1.21) |  | 0.96 (0.79, 1.15) |
| **Community-level variables** | |  |  |  |
| Residence | Urban |  | Ref. | Ref. |
|  | Rural |  | 0.65 (0.53, 0.78) *** | 0.68 (0.56, 0.82) *** |
|  | Nomadic |  | 0.40 (0.31, 0.51) *** | 0.45 (0.35, 0.58) *** |
| Regions | Northwestern Zone |  | Ref. | Ref. |
|  | Northeastern Zone |  | 0.57 (0.46, 0.72) *** | 0.58 (0.47, 0.73) *** |
|  | Southcentral Zone |  | 0.50 (0.42, 0.61) *** | 0.49 (0.40, 0.59) *** |

**p-value < 0.05, ** p-value <0.01, ***p-value < 0.001. AOR: Adjusted Odds Ratio. CI: Confidence Interval, ANC: Antenatal care, PNC: Postnatal care visit. Model 1 (null model) is without independent variables and not specified here.*

Supplementary Table 4. Multilevel mixed-effect binary logistic regression analysis of factors associated with PNC service use among women in Somalia (n = 5,092)

| **Variables** | **Categories** | **Model 2**  **AOR (95% CI)** | **Model 3**  **AOR (95% CI)** | **Model 4**  **AOR (95% CI)** |
| --- | --- | --- | --- | --- |
| **Individual-level variables** | |  |  |  |
| Age of women (years) | 15-20 | Ref. |  | Ref. |
|  | 20–29 | 2.34 (1.54, 3.55) *** |  | 2.24 (1.48, 3.40) *** |
|  | 30–39 | 2.92 (1.82, 4.68) *** |  | 2.63 (1.64, 4.22) *** |
|  | 40–49 | 3.18 (1.62, 6.25) ** |  | 2.82 (1.43, 5.56) ** |
| Educational attainment of women | No education | Ref. |  | Ref. |
|  | Primary | 1.89 (1.48, 2.42) *** |  | 1.83 (1.43, 2.34) *** |
|  | Secondary/above | 2.57 (1.81, 3.66) *** |  | 2.60 (1.83, 3.69) *** |
| Marital status of the mother | Currently Married | Ref. |  | Ref. |
|  | Divorced | 0.75 (0.48, 1.16) |  | 0.79 (0.51, 1.23) |
|  | Widowed | 1.90 (0.95, 3.81) |  | 1.98 (0.99, 3.98) |
| Wealth index | Lowest | Ref. |  | Ref. |
|  | Low | 1.34 (0.86, 2.10) |  | 1.53 (0.97, 2.39) |
|  | Middle | 1.97 (1.27, 3.06) ** |  | 2.34 (1.49, 3.66) *** |
|  | High | 3.36 (2.22, 5.08) *** |  | 3.82 (2.51, 5.82) *** |
|  | Highest | 5.49 (3.59, 8.39) *** |  | 5.83 (3.80, 8.95) *** |
| Media access | Not at all | Ref. |  | Ref. |
|  | < Once a week | 1.34 (0.93, 1.94) |  | 1.39 (0.97, 2.01) |
|  | ≥ Once a week | 1.87 (1.40, 2.49) *** |  | 1.79 (1.34, 2.39) *** |
| Owns a mobile telephone | No | Ref. |  | Ref. |
|  | Yes | 1.46 (1.03, 2.06) * |  | 1.58 (1.11, 2.24) * |
| Parity | Primiparous | Ref. |  | Ref. |
|  | Multiparous | 0.49 (0.37, 0.67) *** |  | 0.51 (0.38, 0.68) *** |
|  | Grand multiparous | 0.34 (0.24, 0.48) *** |  | 0.36 (0.25, 0.52) *** |
| Women empowerment | No | Ref. |  | Ref. |
|  | Yes | 1.44 (1.09, 1.88) ** |  | 1.39 (1.00, 1.82) |
| **Community-level variables** | |  |  |  |
| Residence | Urban |  | Ref. | Ref. |
|  | Rural |  | 0.76 (0.57, 1.02) | 0.77 (0.59, 1.02) |
|  | Nomadic |  | 0.46 (0.30, 0.69) *** | 0.49 (0.32, 0.75) ** |
| Regions | Northwestern Zone |  | Ref. | Ref. |
|  | Northeastern Zone |  | 0.57 (0.40, 0.80) ** | 0.67 (0.48, 0.94) * |
|  | Southcentral Zone |  | 0.48 (0.36, 0.64) *** | 0.56 (0.42, 0.74) *** |

**p-value < 0.05, ** p-value <0.01, ***p-value < 0.001. AOR: Adjusted Odds Ratio. CI: Confidence Interval, ANC: Antenatal care, PNC: Postnatal care visit. Model 1 (null model) is without independent variables and not specified here.*

Supplementary Figure 1. Wealth-related inequalities of ANC utilisation

Supplementary Figure 2. Wealth-related inequalities of early PNC visit
